# Inhibition of Melanization by Kojic Acid Promotes Cell Wall Disruption of the Human Pathogenic Fungus *Fonsecaea* sp.

**DOI:** 10.3390/pathogens11080925

**Published:** 2022-08-17

**Authors:** Jorge Augusto Leão Pereira, Lienne Silveira de Moraes, Chubert Bernardo Castro de Sena, José Luiz Martins do Nascimento, Ana Paula D. Rodrigues, Silvia Helena Marques da Silva, Edilene O. Silva

**Affiliations:** 1 Laboratory of Structural Biology, Institute of Biological Sciences, Federal University of Pará, Belém 66075-110, PA, Brazil; 2 National Institute of Science and Technology in Structural Biology and Bioimaging, Rio de Janeiro 21040-900, RJ, Brazil; 3 Pharmaceutical Sciences Post Graduation Program, Health and Biological Sciences Department, Federal University of Amapa (UNIFAP), Macapá 68903-329, AP, Brazil; 4 National Institute of Science and Technology in Neuroimmunomodulation (INCT—NIM), Rio de Janeiro 21040-900, RJ, Brazil; 5 Laboratory of Molecular and Cellular Neurochemistry, Institute of Biological Sciences, Federal University of Pará, Belém 66075-110, PA, Brazil; 6 Laboratory of Electron Microscopy, Evandro Chagas Institute, Ministry of Health, Belém 66093-020, PA, Brazil; 7 Laboratory of Superficial and Systemic Mycoses, Evandro Chagas Institute, Department of Mycology and Bacteriology, Ministry of Health, Ananindeua 67030-000, PA, Brazil

**Keywords:** chromoblastomycosis, *Fonsecaea* sp., kojic acid, melanin, cell wall

## Abstract

Chromoblastomycosis (CBM) is a chronic human subcutaneous mycosis caused by various aetiologic agents. CBM does not have an established treatment but may be managed using antifungal agents, surgical removal of the lesions, or cryotherapy. Kojic acid (KA), a known tyrosinase inhibitor with a variety of biological actions, including fungistatic action against the fungus Cryptococcus neoformans, mediated by inhibiting melanin production, seems to be an alternative to improve the treatment of CBM. The aim of the present study was to analyze the action of KA against the pathogenic fungus Fonsecaea sp., an aetiological agent of CBM. The fungal culture was incubated with KA, and the amount of melanin was assessed, followed by cytochemical detection. Subsequently, the samples were analyzed by light microscopy, transmission and scanning electron microscopy. Culture analysis revealed that 100 g/mL KA significantly decreased the melanization of the fungus and the exocytosis of melanin into the culture supernatant. Additionally, KA induced less growth of biofilm formation and intense disruption of the cell wall, and decreased the number of melanin-containing vesicles in the culture supernatant. Finally, KA inhibited fungal filamentation in culture and the subsequent phagocytosis process. Thus, KA may be a promising substance to help in the treatment of CBM.

## 1. Introduction

Chromoblastomycosis (CBM) is a subcutaneous fungal infection that primarily affects human hosts, occurring primarily in tropical and subtropical regions [1,2,3]. This infection is caused by pigmented yeast-like fungi that are acquired through the traumatic inoculation of fungal elements into the subcutaneous tissue, causing multiple cutaneous and subcutaneous lesions with verrucous, nodular and plaques morphologies [2,4,5]. This mycosis is caused by different aetiological agents, including *Fonsecaea* sp. In injured tissues, these fungal elements transform into muriform cells with multiplanar septae and are responsible for the establishment of chronic infections [5,6].

One of the main virulence factors of the CBM agent is melanin, a polymer that has been widely described as an important virulence factor in several fungi [7,8,9,10]. This pigment is related to survival in inhospitable environments and to antifungal therapy resistance [11,12,13,14]. Two main pathways are well known to synthesize melanin in fungi: 1,8-dihydroxynaphthalene (DHN) and L-3,4-dihydroxyphenylalanine (L-DOPA) [11]. In the DHN pathway, acetyl-CoA or malonyl-CoA are used by polyketide synthase to produce 1,3,6,8-tetrahydroxynaphthalenine (1,3,6,8-THN) following the production of DHN molecules, which are polymerized to produce DHN melanin [15,16]. In the L-DOPA pathway, L-DOPA is used by laccase to produce dopaquinone and dopachrome, which are polymerized to produce L-DOPA melanin [11]. Mammalian melanin biosynthesis occurs in a similar manner to the L-DOPA pathway in fungi, also using L-DOPA or tyrosine to produce dopaquinone but using the enzyme tyrosinase. Laccase and tyrosinase are copper-containing enzymes, with two types of copper atoms in their active site, able to catalyze the oxidation of phenolic compounds [11,17].

CBM does not have an established treatment but may be addressed through the use of antifungal agents, surgical removal of the lesions, or cryotherapy [18,19,20,21,22]. The antifungals used for treatment have some serious side effects, such as high nephrotoxicity and hepatotoxicity [23,24]. The continuous use of these antifungal drugs can also lead to drug resistance [25,26]. The search for new compounds with antifungal action is needed to improve the treatment of these infections. Kojic acid (KA), a secondary metabolite produced by fungi of the genera Aspergillus and Penicillium [27,28], has numerous applications, being used primarily as a tyrosinase inhibitor [29,30], as an antioxidant agent, and as a food additive [31,32]. In addition, KA has fungistatic activity [33], induces the activation and differentiation of immune system cells [34,35,36], and acts against the parasite *Schistosoma mansoni* [37], as well as against the protozoan *Leishmania (Leishmania) amazonensis* [38]. Furthermore, in filamentous fungi, KA appears to act synergistically with conventional antifungal drugs, potentiating their action [39]. Thus, the present study was undertaken to analyze the action of KA on a clinical isolate of CBM in vitro.

## 2. Materials and Methods

### 2.1. Obtaining, Maintenance, and Cultivation of Clinical Isolate

**Figure 1:**
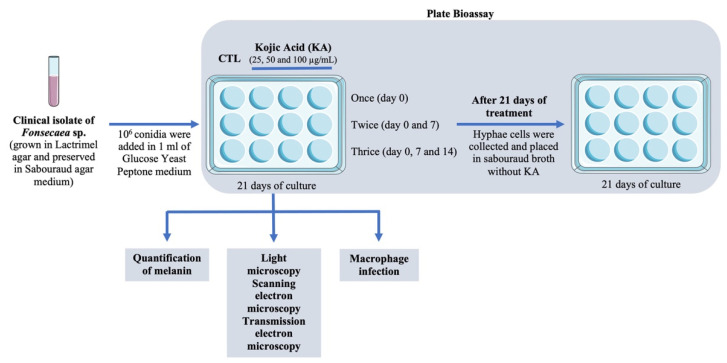
Experimental design. Conidial cells of *Fonsecaea* sp. from one clinical isolate were cultivated to perform a plate bioassay during treatment with kojic acid (KA). To analyze the role of KA on *Fonsecaea* sp., melanin was quantified, and the morphology of the fungal cells was observed by light microscopy and scanning and transmission electron microscopy. Conidia cells were also used to analyze the role of KA during the infection of murine macrophage host cells. As a control, untreated conidia cells and untreated infected macrophages were used.

The clinical isolate of *Fonsecaea* sp. used in this study was obtained in 2006 from a human case with a desquamative lesion and long-standing chromoblastomycosis. The culture was kept by the Mycology Laboratory of the Evandro Chagas Institute (Belém, Pará, Brazil) and maintained on Sabourad dextrose agar for a continuous period of 21 days. Each culture containing a 1 cm^2^ fragment was observed and identified following the microculture method in slides [40]. To obtain *Fonsecaea* sp. hyphae containing conidia, the mycelial form was inoculated in broth for seven days at 30 °C. To separate hyphae from conidia, the cell suspension was placed in a tube with 10 mL of 0.45% NaCl, vortexed for one min, filtered on filter paper and centrifuged at 1500 rpm for one min to release the conidia to be used in the experiments, as shown in Figure 1.

### 2.2. Obtaining and Diluting Drugs

Kojic acid (KA) and tricyclazole (TZL) were obtained from Sigma-Aldrich Co. (St. Louis, MO, USA). A stock solution was prepared at a final concentration of 1 mg/mL in distilled water, and further dilutions were made from this solution.

### 2.3. Plate Bioassay

To test the antifungal activity of KA, the methods were performed as described previously with some modifications [41,42]. In brief, 10^6^ conidia were added to 1 mL of glucose yeast peptone medium (20 g/L glucose, 5 g/L peptone, and 5 g/L yeast extract) containing KA (12.5, 25, 50 and 100 µg/mL) or medium alone as a control. The conidia were incubated at room temperature, and each experimental group was treated once (Day 0), twice (Days 0 and 7), or thrice (Days 0, 7, and 14) and analyzed after 21 days of treatment. To determine if the fungal culture recovered growth after the treatment period, aliquots of cultures in the filamentous phase were collected and placed in Sabouraud broth without KA and maintained under the same conditions as used before.

### 2.4. Microscopy Analysis

The 21-day KA-treated and untreated fungal cultures (control group) were processed for light microscopy (LM), scanning electron microscopy (SEM) and transmission electron microscopy (TEM). For LM, the cells were fixed with 4% paraformaldehyde in 0.1 M PHEM buffer (MgCl_2_-5 mM; KCl-70 mM; EGTA-10 mM; HEPES-20 mM; and PIPES-60 mM), pH 7.2, for 1 h at room temperature. The cells were washed to identify hyphal spreading during examination with an Axio scope A1 Zeiss microscope. For SEM, cells were fixed with 1% glutaraldehyde, 4% paraformaldehyde and 2.5% saccharose in 0.1 M PHEM buffer, pH 7.2, for 1 h at room temperature, and then postfixed in 2.5% glutaraldehyde, 4% paraformaldehyde, and 2.5 M calcium chloride in 0.1 M cacodylate buffer, pH 7.2, for 1 h. The cells were washed and postfixed again in 1% osmium tetroxide and 1% ferrocyanide for 1 h, dehydrated in graded ethanol, critical point dried (CO_2_ in air), coated with gold, and examined with an LEO 1450 VP SEM. For TEM, the cells were fixed with glutaraldehyde (2.5%) and paraformaldehyde (4%) in cacodylate buffer (0.1 M) for 1 h at room temperature, postfixed in osmium tetroxide solution (1%) and ferrocyanide (0.8%), dehydrated in graded acetone, and embedded in epoxy resin. Ultrathin sections were obtained with an ultramicrotome (EM UC6-S6E, Leica, Wetzlar, Germany), stained with uranyl acetate/lead citrate, and examined with a TEM (EM900, Zeiss, Jena, Germany).

### 2.5. Cytochemical Detection and Quantification of Melanin

Cultures treated three times with 100 µg/mL KA were filtered to separate the fungal mass (0.5 g) and the supernatant. The supernatant was ultracentrifuged at 15,000 g ( CP70MX, Hitachi, Tokyo, Japan) to obtain vesicles according to a previously published procedure [43]. The cells and vesicles were processed with the Fontana–Masson stain, a cytochemistry method that is specific for the detection of melanin [44,45]. The fixation was carried out with 1% glutaraldehyde for one hour, and the samples were then washed with distilled water, incubated with ammonium silver nitrate solution (10%) for 30 min, washed in distilled water, fixed again with 1% glutaraldehyde and 4% paraformaldehyde, washed again with distilled water, dehydrated in graded acetone, and embedded in epoxy resin. The ultrathin sections were made using an ultramicrotome (Leica EMUC6) and examined with a Zeiss EM900 TEM. For total melanin quantification [45], melanin was initially measured in the fungal mass. Vesicles released from mass fungi were also quantified. These vesicles were centrifuged at 100,000 g for min at 4 °C to separate the pellet from the supernatant. After that, the pellet and supernatant were weighed and solubilized in 1 N NaOH and 10% DMSO to extract the melanin content at 80 °C in a thermo shaker (VHD-B1-AQ/AG-100) for one hour. After melanin extraction, the samples were cooled and centrifuged at 3500 rpm for 15 min to measure the amount of melanin at 490 nm with a spectrophotometer (EL×800, BioTek, Beijing, China). As a positive control for inhibiting melanization, one additional culture group was supplemented with a final concentration of 16 µg/mL tricyclazole, which is an enzyme inhibitor of melanin synthesis in dematiaceous fungi.

### 2.6. Analysis of Vesicles in the Culture Supernatant

To quantify and analyze the morphology of extracellular vesicles, 150 mL culture-conditioned medium of 21-day liquid-treated culture with KA was used. To obtain cell-free vesicles, the medium was centrifuged (once at 15,000 g and twice at 100,000 g at 4 °C for one hour each) to obtain the final precipitate. Each precipitated sample was fixed with 1% glutaraldehyde in 0.1 M PHEM buffer, pH 7.2, and processed with Fontana–Masson stain as described previously. The vesicles were analyzed with a Zeiss EM900 TEM and quantified using ImageJ software 1.52a. Untreated cultures were used as the control group.

### 2.7. Cell Differentiation Analysis in KA-Treated Fungal Cultures and during Host Cell-Fungal Interactions

To test the activity of KA on the differentiation of fungal cultures, 10^6^ conidia were added to the coverslip to be cultured in 1 mL of DMEM containing 100 µg/mL KA or medium alone. After 24 h of incubation at 37 °C and 5% CO_2_, the cells were stained with 10% Giemsa, and the filamentous forms were quantified from 100 total conidia cells. The size of the cells was measured by ImageJ software. To analyze the intracellular fungal filamentation in infected KA-treated macrophages following phagocytosis, murine macrophages were obtained from peritoneal cavities of Mus musculus Balb/c mice with DMEM, pH 7.2. After one hour of incubation at 37 °C and 5% CO_2_, the nonadherent cells were washed away with PBS, pH 7.2, and adherent macrophages were incubated overnight in DMEM supplemented with 10% heated-inactivated fetal bovine serum (FBS). The animals were euthanized in accordance with the committee of animal research and ethics of the Federal University of Pará N°. 8482280916. After that, the interaction of conid cells with macrophages was performed for 3 h of incubation (MOI of 5:1), and infected cells were washed three times with PBS, pH 7.2, and treated with 100 µg/mL KA for 24 h. After that, the cells were fixed with 4% paraformaldehyde and stained with 10% Giemsa.

### 2.8. Statistical Analysis

All experiments were performed in triplicate. The mean and standard deviation of the three independent experiments were determined, and the data were analyzed using GraphPad Prism 6.0 (GraphPad Software, La Jolla, CA, USA), followed by an analysis of variance (ANOVA) and the Tukey test. A *p*-value of less than 0.05 was considered statistically significant.

## 3. Results

### 3.1. Effect of KA Action on the Melanization of Chromoblastomycosis Agent

**Figure 2:**
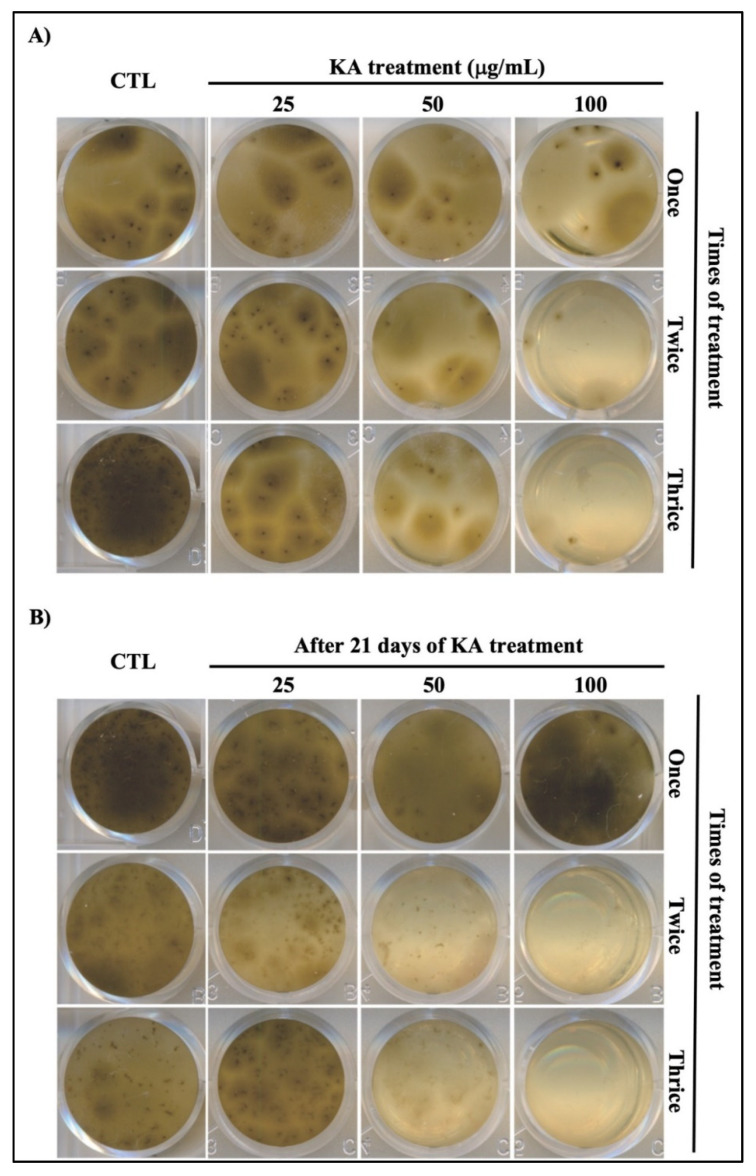
Plate bioassay showing KA action on melanization. (**A**) Cultures were treated with KA once (Day 0), twice (Day 0 and Day 7) or thrice (every seven days) for 21 days and compared with untreated control cultures. Note that more than one treatment showed a great reduction in melanization. (**B**) Aliquots of each culture after 21 days of KA treatment were replaced in Sabouraud broth without KA and maintained under the same conditions for more 21 days. Note that the cultures with altered melanization did not recover the melanization processes.

The cultures treated with different concentrations of KA (Figure 2) showed an apparent decrease in mycelial growth in a dose- and treatment-dependent manner. The reduction in mycelial growth was accompanied by a decrease in dark staining, mainly in the cultures that received three treatments (Days 0, 7 and 14), when compared to the control group without treatment (CTL) (Figure 2A).

**Figure 3:**
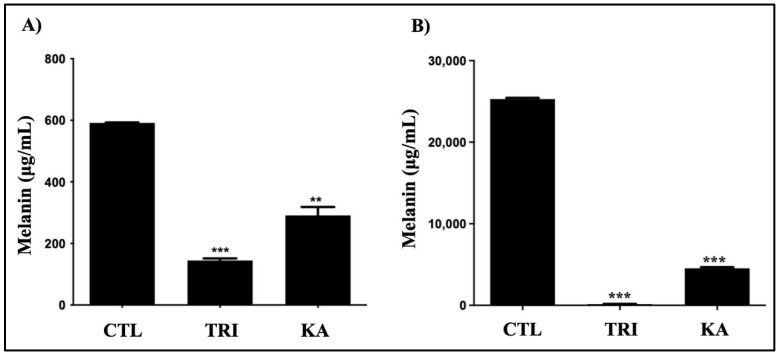
Production of melanin. Melanin was quantified in fungus cells (**A**) and supernatants (**B**) of cultures: control (CTL) and tricyclazole (TRI: 16 μg/mL) or kojic acid (KA: 100 μg/mL)-treated cells. The data represent the mean of three separate experiments, and error bars are +/− SEM, followed by an analysis of variance (ANOVA) and the Tukey test (***), *p* < 0.001 and (**), *p* < 0.01.

To analyze the possible recovered growth and melanization after KA treatment, aliquots of each culture were removed and maintained under the same conditions but without KA treatment. The once-treated cultures were able to recover their dark color at all the concentrations evaluated, whilst the cultures that received two or three treatments did not recover their growth or dark color (Figure 2B). To determine where melanin was retained, a cytochemical assay was performed (Fontana–Masson) to separately quantify the melanin content of the fungal cell mass and extracellular medium containing the released vesicles. As shown in Figure 3A,B, when compared to untreated control cells, melanin was decreased in fungus cells (A) and supernatants (B) after treatment with KA and tricyclazole, a known melanin synthesis inhibitor. These data confirm the action of KA on the inhibition of melanization by this fungal agent.

### 3.2. Microscopy of Chromoblastomycosis Agent after KA Treatment

**Figure 4:**
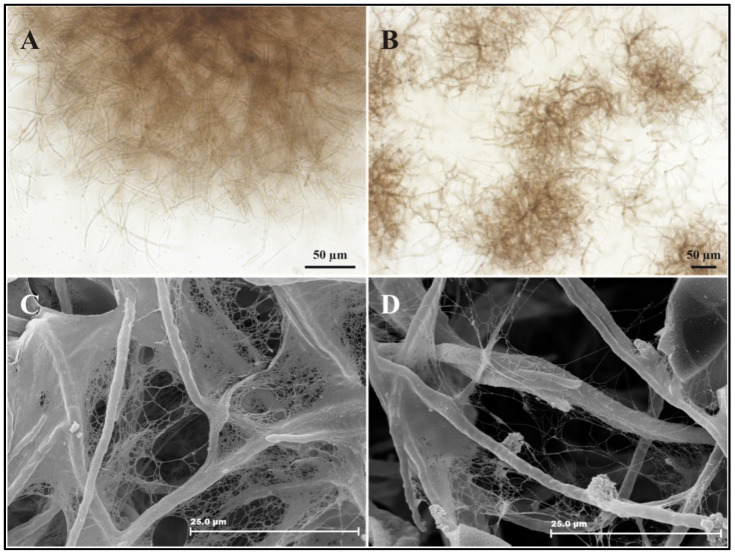
Morphological analysis of CBM agents after KA treatment. Control (**A**,**C**) and KA-treated cells (**B**,**D**) were observed by light microscopy (**A**,**B**) and scanning electron microscopy (**C**,**D**) after 21 days of culture. Note that KA treatment reduced biofilm formation.

Light microscopy analysis of untreated (Figure 4A,C) and 100 μg/mL KA-treated cultures (Figure 4B,D) showed that, after 21 days, the KA-treated cultures had many more cells spread out compared to cells in untreated control cultures. SEM analysis showed that KA treatment reduced biofilm formation (Figure 4D), consistent with the spreading out of cells in liquid culture.

### 3.3. Quantification of Externalized Vesicles

**Figure 5:**
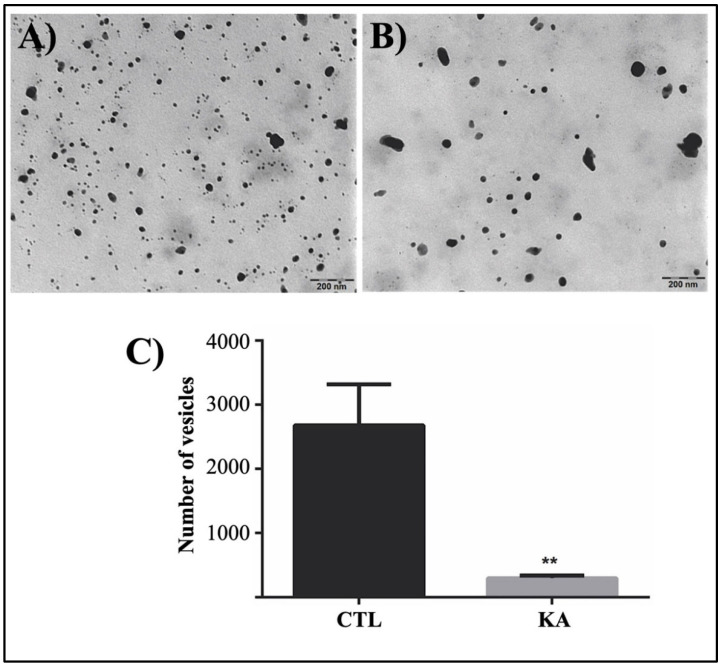
Quantification of vesicles in the culture supernatant. The supernatants of control cells (**A**) and KA-treated cells (**B**) were observed by transmission electron microscopy after 21 days of culture. The quantification of vesicles (**C**) shows a significant decrease after treatment with KA. Data represent the means of three separate experiments, and error bars are +/− SEM, followed by an analysis of variance (ANOVA) and the Tukey test (**), *p* < 0.01.

To verify whether the decrease in melanization in the culture supernatant was related to the number of externalized vesicles, quantification of these supernatant vesicles was carried out (Figure 5). Interestingly, a significant reduction in the number of vesicles (Figure 5B) in free supernatant cells in the cultures treated with 100 μg/mL KA was observed as compared to those in the control group (Figure 5A). The low number of these vesicles in the supernatant corroborates the biological action of KA in fungal melanization as a melanin synthesis inhibitor, decreasing the accumulation of melanin in the fungal cell and supernatant.

### 3.4. TEM Analysis

**Figure 6:**
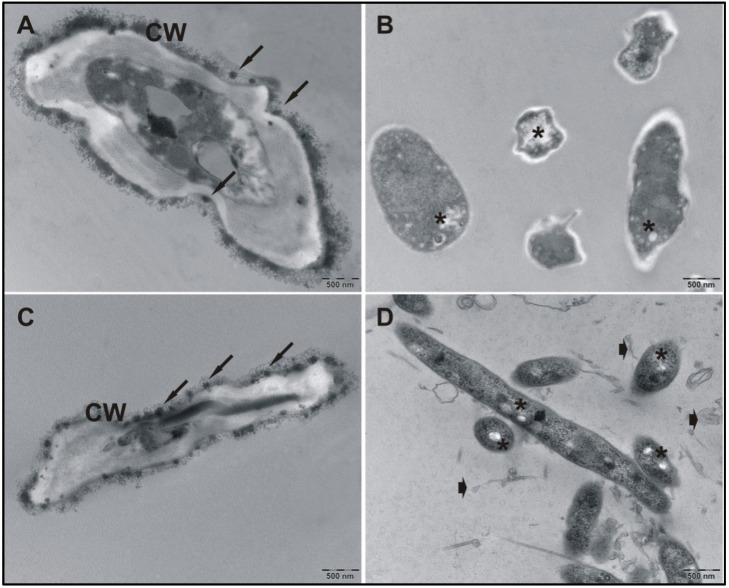
TEM analysis of KA action on the CBM agent. (**A**,**C**) Control cultures; (**B**) cultures treated with 100 μg/mL KA; and (**D**) cultures maintained after treatment. Control cells maintained an integrated cell wall (CW) and retained electron-dense granules (arrows). Treated cells maintained after treatment presented intense cell wall disruption and the presence of electron-lucent granules in the cytoplasm (*) and cellular debris (arrowheads).

Ultrastructural analyses were performed to determine possible cell-wall alterations that occurred after treatment with 100 μg/mL KA. The treatment induced an intense disruption of the fibrillar portion of the cell wall (Figure 6B) as compared to that of the untreated control group (Figure 6A,C). In addition to this disruption, intracytoplasmic accumulation of electron-lucent vesicles was observed (Figure 6B). The treated fungal cells that were maintained posttreatment did not recover their cell wall structure, and numerous cellular debris particles and electron-lucent granules characteristic of nonviable cells were present (Figure 6D). The untreated control cells exhibited normal characteristics of cell wall morphology, with the presence of electron-dense granules.

### 3.5. Action of KA on Fungal Filamentation in Culture and During Fungus-Macrophage Interaction

**Figure 7:**
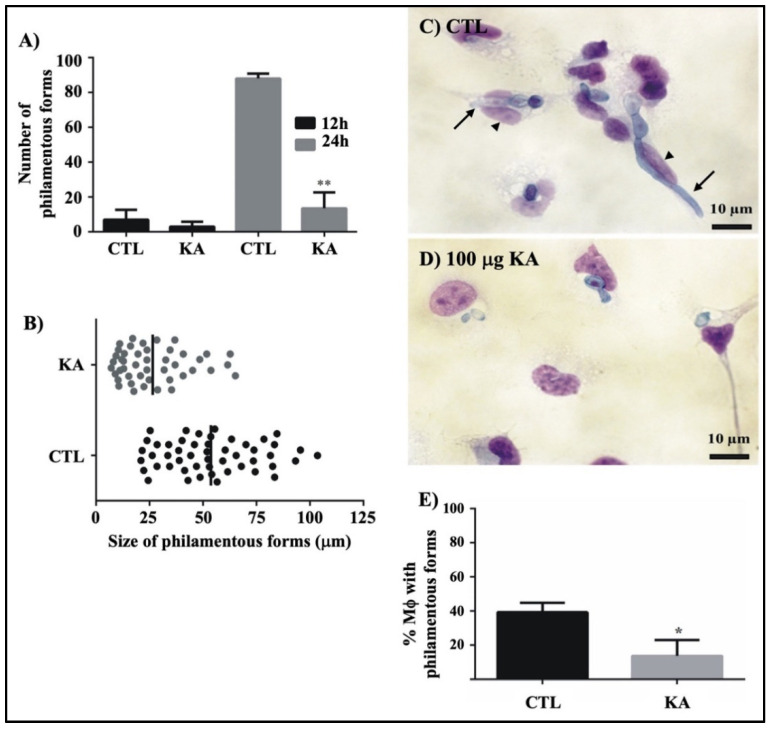
Kojic acid action on fungal filamentation. Quantification (**A**) and size (**B**) of hyphae during 24 h of culture, (**C**) untreated infected macrophages (CTL) with the intracellular formation of hyphae, and (**D**) treated infected macrophages (100 μg/mL) with conidia after 24 h of infection. (**E**) Percentage of macrophages with filamentous forms. Note that treatment with KA affects fungal filamentation in culture and during host-cell interactions. Arrows: fungal cells; arrowheads: macrophage cells. Data represent the means of three separate experiments, and error bars are +/− SEM, followed by an analysis of variance (ANOVA) and the Tukey test (*) *p* < 0.05 and (**), *p* < 0.01.

To verify the possible relationship between cell-wall disruption after KA treatment and the development of hyphae, a temporal analysis was performed during 24 h of culture. We observed that treatment with 100 μL/mL KA significantly reduced the number (Figure 7A) and size of hyphae (Figure 7B) after 24 h of culture.

We also analyzed intracellular fungal filamentation following phagocytosis of conidia by the host cell. As shown in Figure 7D, treatment with 100 μL/mL KA inhibited hyphal growth inside macrophages and, as shown in Figure 7E, we observed that the percentage of macrophages with filamentous forms decreased as compared to the control.

## 4. Discussion

Chromoblastomycosis (CBM) is a disease caused by pigmented yeast-like fungi (melanin producers) that are involved in fungal pathogenesis [46]. In *Fonsecaea pedrosoi*, which is the primary etiological agent of CBM, melanin is deposited in the cell wall and is directly related to the protection of the fungal cell against stressful environments and the host immune response [47,48,49]. However, the effects of KA on *Fonsecaea* sp., a potent inhibitor of the enzyme tyrosinase, which is involved in the synthesis of melanin, are not well known [50,51]. Thus, in the present study, we investigated whether KA affects the pathogenic fungus *Fonsecaea* sp., an aetiologic agent of CBM.

As the basis for the experimental design of this study, we have used only one clinical isolate of *Fonsecaea* sp, but we also tested another isolate with similar results. Initially, the clinical isolate of *Fonsecaea* sp. was grown in three KA exposure groups and compared with a control group. Each KA-treated group received 25, 50 and 100 μL/mL KA once, twice, or thrice (every seven days for 21 days). Our results demonstrated a reduction in mycelial growth and dark staining in the KA-treated groups compared to in the control group. Additionally, fungal growth was not able to recover after two or three treatments with KA. These data reveal that KA was able to inhibit fungal growth and suggest that melanin production and/or other melanin-dependent properties of *Fonsecaea* sp. might also be defective.

Filamentous fungi grow by hyphal extension through the exocytosis of vesicles, which are carried by microtubules to the apical part of the hyphae (polarized exocytosis), delivering growth supplies, including melanin, membranes, and cell wall synthase proteins [52]. This hyphal extension overcomes long distances and is related to the invasive capacity of fungi to the substrates, either in the saprophytic or host tissue environment [52,53]. Apical growth is mediated by proteins involved in the synthesis and transport of components that form the membrane and the fungal cell wall [52,54]. The decrease in hyphal growth promoted by KA treatment may be related to the interference in vesicular transport or to the inhibition of enzymes, such as chitin synthase and glucan synthase or tyrosinase, which are responsible for the synthesis of cell wall constituents and the elongation of hyphae.

After spectrophotometry, quantification of intracellular and extracellular melanin confirmed that KA treatment inhibited the production of melanin. The observed decrease in melanin production may be related to the action of KA on vesicle traffic since there are no reports that this substance has any action on polyketide synthase, a group of enzymes responsible for the production of melanin in CBM agents [11,55]. However, we cannot rule out the hypothesis that KA inhibits melanin synthesis via the DHN and/or L-DOPA pathway, as is achieved by tricyclazole and most other melanin biosynthesis inhibitors [15,55,56].

A study on *Cryptococcus neoformans* revealed that sizes of extracellular vesicles are heterogeneous and also related to melanization. Smaller diameter vesicles are associated with melanization and larger diameters are associated with non-melanization [47]; i.e., the amount of melanin seems to be inversely proportional to the size of vesicles. This may explain why KA-treated cells secreted only large vesicles when extracellular vesicles were quantified. The cell wall is a structure that confers fungal protection against osmotic pressure and environmental factors and provides storage of vesicles containing substances with important biological functions, such as lipids, proteins, polysaccharides, and melanin, which interact directly with the external environment and help to modulate the immune response of the host [47,54]. It is known that the cell-wall components of *Fonsecaea* sp. hyphae modulate the host immune response, and this factor may be involved in fungal survival [57]. Another study has shown that cell wall alterations induce important effects on fungal physiology [58]. Our SEM and TEM results demonstrated that KA induced many morphological changes in the fungi, such as decreased biofilm accumulation, disrupted cell walls, and the accumulation of intracytoplasmic electron-lucent vesicles.

The hypothesis that vesicle traffic may be affected by KA treatment may also explain the disruption of the cell wall since the constituents of this structure, such as chitin and β-glucans, are synthesized in the cytosol of fungal cells and then transported to the surface, restructuring the cell wall [11]. Melanin is also known to form covalent bonds with polysaccharides, important constituents of the cell wall [11]. Tricyclazole, a specific DHN-melanin inhibitor, is known to induce the accumulation of vesicles in the fungal cytoplasm, demonstrating that the decrease in melanin production inhibits vesicular traffic since the externalization of the vesicle is induced by cell wall disruption [55].

Furthermore, it has been reported that melanin is an important factor in virulence due to its antioxidative and metal-binding properties [59]. It appears that melanin reacts with ferric iron to reduce it to ferrous iron and maintains an iron-melanin complex as a redox buffer [60]. KA is a known metal chelator [61,62], and we hypothesize that KA may target the melanin/metal association, thereby inducing alterations to the physiology of this fungus with modifications to the structure and organization of the cell wall. A further possibility is that KA increases the production of free radicals, which interferes with the redox potential of melanin, thereby changing its structure and organization.

Another important finding of our research was that KA treatment also inhibited cellular filamentation in fungal culture and during macrophage infection. These data suggest that KA also acts as a fungistatic drug. Not only is cell-wall melanin known to be an evasion mechanism but so too is fungal filamentation. The internalization of pathogenic fungi by macrophages induces the transition to filament forms that promote macrophage death to save the fungus from killing by immune cells [12,13,63]. Filamentous fungi are known to form hyphae inside host cells, challenging macrophages because of the extreme length of the hyphae [64]. Another important aspect after KA treatment is the inhibition of melanin production, which can also facilitate phagocytosis. Melanized fungi are well known to enhance resistance to phagocytosis by macrophage cells, modifying the complement system and antibody responses, and reducing the effectiveness of antifungal drugs [11,65]. In some species of fungi, vesicle-associated melanization is related to cell wall biosynthesis and is known as an important virulence factor [47]. Vesicle retention in the cell wall has been described in different species of pathogenic fungi, including *C. neoformans* and *Candida albicans* [53,66]. Melanin-deficient fungi are well known to show attenuated virulence, are unable to prevent complement activation, neutralize antimicrobial peptides, or protect against reactive oxygen species (ROS) [11,67].

## 5. Conclusions

In conclusion, our results demonstrate for the first time that KA has an important action in vitro on a clinical isolate of a chromoblastomycosis agent, promoting inhibition of fungal growth, intense disruption of the cell wall with vesicle externalization, reducing biofilm formation, decreasing melanization, and affecting fungal filamentation in culture and during phagocyte interaction. Thus, considering the crucial role of the cell wall and the production of melanin for fungal survival, KA may have potential therapeutic application in the treatment of chromoblastomycosis.

## Data Availability

Not applicable.
